# Conceptualizing the transfer of knowledge across cases in transdisciplinary research

**DOI:** 10.1007/s11625-017-0444-2

**Published:** 2017-06-12

**Authors:** Carolina Adler, Gertrude Hirsch Hadorn, Thomas Breu, Urs Wiesmann, Christian Pohl

**Affiliations:** 1 Environmental Philosophy Group, and Transdisciplinarity Laboratory (D-USYS TdLab), Institute for Environmental Decisions, ETH Zurich, CHN H 73.1, Universitaetstrasse 16, 8092 Zurich, Switzerland; 2 Environmental Philosophy Group, Institute for Environmental Decisions, ETH Zurich, CHN H 73.2, Universitaetstrasse 16, 8092 Zurich, Switzerland; 3 Centre for Development and Environment (CDE), University of Bern, Hallerstrasse 10, 3012 Bern, Switzerland; 4 Department of Integrative Geography, Institute of Geography, University of Bern, Hallerstrasse 10, 3012 Bern, Switzerland; 5 Transdisciplinarity Laboratory (D-USYS TdLab), Institute for Environmental Decisions, ETH Zurich, CHN K 78, Universitaetstrasse 16, 8092 Zurich, Switzerland

**Keywords:** Case study, Evidence-based policy, Transferability, Transdisciplinary research, Quality, Scalability

## Abstract

Transdisciplinary (TD) research is increasingly suggested as a means of tackling wicked problems by providing knowledge on solutions that serve as pathways towards sustainable development. In contrast to research striving for generalizable findings, TD research produces insights for a particular case and context. TD researchers, who build on other TD projects’ results, need to know under what conditions knowledge gained from their case can be transferred to and applied in another case and context. Knowledge transfer between researchers and stakeholders is extensively discussed in the literature. However, a more profound understanding and management of the challenges related to knowledge transfer across cases, as it applies to TD research, are missing. We specify the challenges of knowledge transfer in TD research by distinguishing TD research for policy from conventional evidence-based policy, which relies on generalizing findings, such as randomized controlled trials. We also compare the functions that cases fulfil in other types of research that include basic, applied and ideographic research. We propose to conceptualize transferability of knowledge across cases as arguments by analogy. Methodologically, this would imply explicit consideration on whether the cases in question are sufficiently similar in relevant aspects while not dissimilar in other additional relevant aspects. On the one hand, this approach calls for explicit material considerations that are needed to learn about which aspects of cases are relevant. On the other hand, formal considerations on how to weigh perceived relevant similarities and dissimilarities of the cases at hand for transferability of knowledge, are needed. Empirical research on how projects in TD research deal with this problem is called for.

## Introduction

Research for sustainable development deals with wicked problems in society by generating knowledge on the multiple processes of change, such as global environmental change, where numerous dynamic exchanges in human-environment systems simultaneously exert impacts and feedbacks into said systems. Dealing with impacts of such interacting processes of change requires: (1) a fundamental understanding of components and dynamics within and between systems (systems knowledge), (2) knowledge to clarify and prioritize the values at stake in dealing with these impacts (target knowledge) and (3) knowledge on how we could transform the systems to account for these values, (transformation knowledge) (adapted from ProClim 1997). All three forms of knowledge might provide insights relevant to policy in dealing with these impacts as solutions that are consistent with long-term sustainable development. In addition, policy relevant research has to make sure it is sensitive to the local context of problems, as is the case in transdisciplinary (TD) case study research. In this paper, we refer to TD research as joint knowledge production of these three forms of knowledge between researchers of different disciplines and stakeholders from society, the private and the public sector (Hirsch Hadorn et al. 2008; Wuelser et al. 2012).

If TD researchers want to build on other TD projects’ results, they need to know under what conditions knowledge produced in one case can be transferred to and applied in another case. While knowledge transfer between researchers and stakeholders, or more generally between science and policy, is extensively discussed in the literature, a profound understanding and management of the challenges related to knowledge transfer across cases are missing. Therefore, we call for urgent and concerted consideration to matters of knowledge transfer and application between cases as a methodological challenge that the TD research community needs to address.

In this paper, we propose a conceptual approach and point at the methodological implications for addressing and assessing knowledge transfer across cases in TD research. In “Framing the problem and current practice”, we start with a brief sketch of the current practice in TD research in order to highlight that transferability across cases is an issue that needs methodological consideration based on an appropriate conceptualization of the problem. We discuss our problem framing on the challenges of transferring knowledge across cases and distinguish different ways of transfer across cases. We then comment on proposals in the literature as a basis for addressing the methodological gap regarding transferability across cases and propose to conceptualize the problem as argument by analogy. In “Shortcomings of the conventional approach to evidence-based policy from a TD perspective”, we show why TD research cannot bypass those challenges of analogical inference by building on generalizable findings from approaches such as randomized controlled trials (RCTs), used in conventional evidence-based policy. In “The specific challenges of transfer of knowledge across cases for TD research”, we clarify the specific challenges of transfer for TD research by comparing it to four other ways of investigating cases. We propose to handle transferability of knowledge from TD case study research across cases with reference to whether the cases in question are sufficiently similar in relevant aspects while not dissimilar in additional relevant aspects (“Methodological implications of conceptualizing transfer of knowledge across cases in TD research as analogical arguments”). This approach includes on the one hand formal considerations and related criteria for how to weigh perceived similarities and dissimilarities against each other for the cases at hand. Here, TD research can build on existing literature in argument analysis as a starting point. On the other hand, material considerations are needed to learn about which aspects of cases are relevant. Here, empirical research on how projects in TD research deal with this problem is called for. In “Summary and conclusion”, we conclude with suggestions to advance case-based methodology in TD research.

## Framing the problem and current practice

A common way of relating research with policy processes is through synthesis reviews. Such reviews assess and synthesize scientific findings from multiple and diverse studies to inform policymakers, for instance, as is the case with boundary organizations like the Intergovernmental Panel on Climate Change (IPCC) and the Intergovernmental Platform on Biodiversity and Ecosystem Services (IPBES). In the IPCC case, scientific evidence informing mitigation and adaptation to climate change is provided through a synthesis of findings derived from models, simulations and observations, ensuring scientific credibility for policymakers who negotiate on targets and measures under the United Nations Framework Convention on Climate Change (UNFCCC). Procedural legitimacy, i.e. accounting for perspectives of policymakers, is ensured via line-by-line approval of the Summary for Policymakers during the IPCC plenaries. Given that efforts to mitigate the effects of climate change appear ineffective, adaptation to the impacts of climate change is gaining urgent importance (Peters et al. 2013). However, in assessing scientific findings on adaptation for policymakers, it is not sufficient to focus only on evidence, decoupled from its policy relevance in context (Rose 2014). Given the importance of local and context-specific factors for effective adaptation, knowledge on ‘what works’ has to rely on diverse and multiple case studies (Brunner 2010). Still, problems of ambiguity and inconsistency arise when numerous and diverse forms of case-specific knowledge are assessed against unspecified or vague criteria to evaluate both the evidence for and the relevance of the knowledge for the problem at hand. Consequently, guidelines issued by the IPCC to its authors to ensure consistency appear inadequate in fulfilling that goal when it comes to the assessment and aggregation of case-specific knowledge (Adler and Hirsch Hadorn 2014). Although no assessment reports have been yet issued by IPBES, deliberations on assessment processes reflect similar concerns (Turnhout et al. 2012). Key in this debate is how to ensure policy-relevant assessment findings when knowledge is based on context-specific cases with diverse disciplinary perspectives (Turnhout et al. 2012).

Another way how research for sustainable development and policy processes inter-relate is through problem-oriented research like policy sciences in the USA (Brunner 2010) and TD research in European countries. In TD research, researchers and policy-makers or stakeholders from administration, civil society and the private sector interact at specific stages during the whole research process, from identifying and framing a problem, analysing it, and bringing solutions to fruition. TD research strives for (a) grasping the relevant complexity of a problem, (b) accounting for multiple and diverse values that underpin diverse perceptions of that problem, (c) linking abstract and case-specific insights to build an understanding of the problem and (d) elucidating options for change based on common interest (Pohl and Hirsch Hadorn 2007; Wiesmann and Hurni 2011).

While assessment procedures of boundary organizations like the IPCC are challenged when aggregating context-specific knowledge on complex cases, TD research is challenged when inferring whether knowledge co-produced for a case is also applicable to another, since both a conceptualization of the problem and a methodology for transfer across cases are missing. Also, it appears that this is not a prominent topic among many other challenges mentioned for conducting TD research (e.g. Jahn and Keil 2015; Lang et al. 2012; Polk 2014). Here, we focus explicitly on outlining key considerations for transferring knowledge developed in one case for application into another case.

We use the term ‘knowledge’ following the customary distinction in TD research between systems knowledge, i.e. a fundamental understanding of components and dynamics within and between systems; target knowledge, i.e. knowledge to clarify and prioritize the values at stake in dealing with impacts; and transformation knowledge, i.e. knowledge on how we could transform the systems to account for these values (adapted from ProClim 1997). These forms of knowledge encompass a broad range of information sources such as scientific knowledge from researchers of different disciplines and expertise, know-how and experience of stakeholders and practice experts from the public and private sector, and civil society. In addition to systems, target and transformation knowledge on the problem at hand, i.e. the substance, TD research also develops knowledge about procedures and processes for how to deal with the range of issues in TD case study research, i.e. methods for co-production of knowledge for doing TD research.

**Fig. 1:**
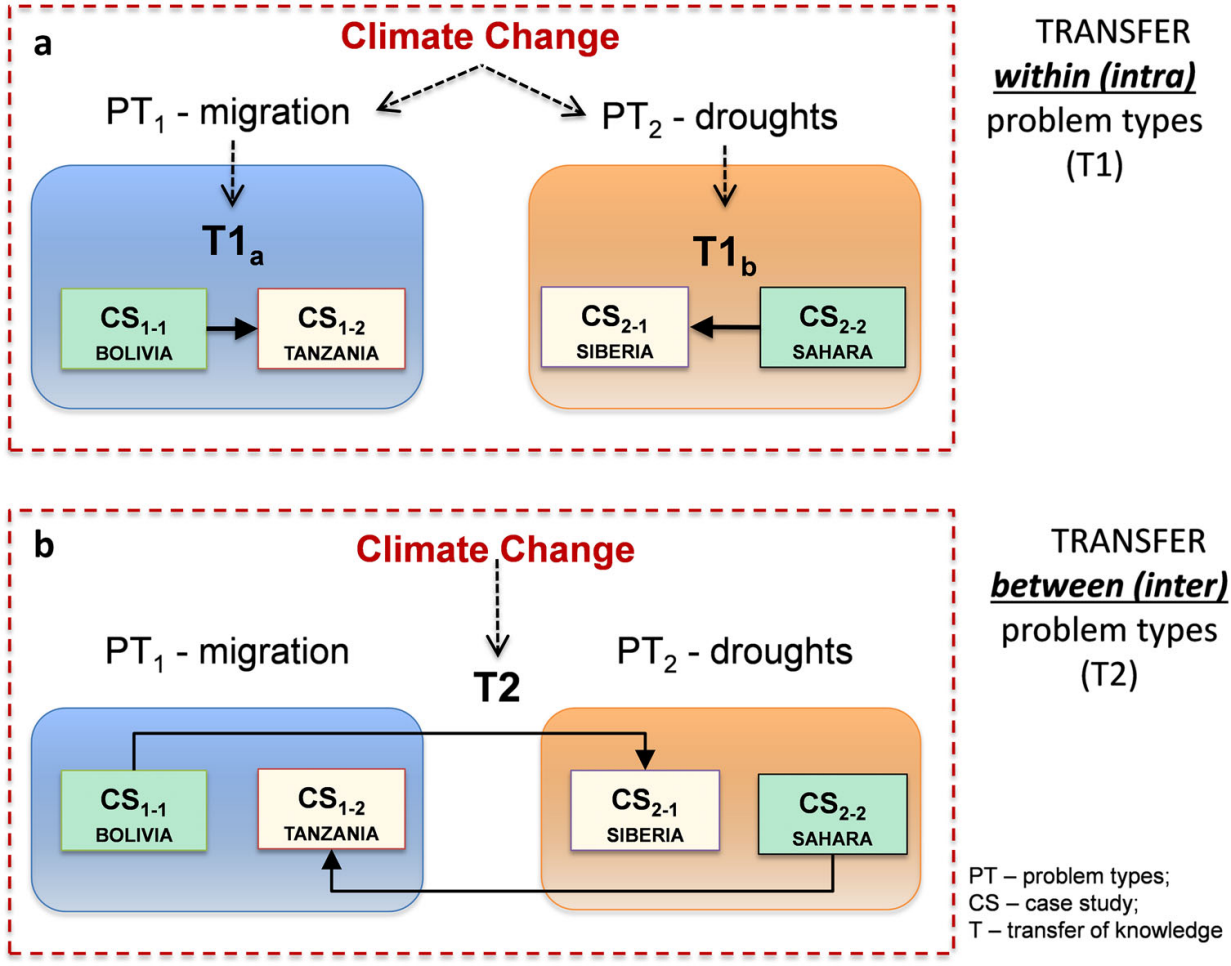
Two ways of transferring knowledge between cases: **a** between units of the same problem type (*T1*); and **b** between units of different problem types (*T2*)

We use the terms ‘transfer of knowledge’ as applying substantive knowledge derived in one context (case), or methods that have been used to study that case, to another case or type of problem. The term ‘transferability’ is used to determine whether such a transfer would be appropriate, which is a normative methodological consideration. Considering transferability of knowledge in TD research is important. For instance, when developing policies based on TD research, the interest is not only on whether they will be effective in the case under investigation, but also whether they will be so in another case. Consider the following examples (see Fig. 1).

We can think of two situations that depict two types of transfer of knowledge from the researched case to the un-researched case. In the first situation a), we hypothetically wish to learn about climate change based on evidence from numerous case studies. We can make this assessment, for example, by focusing on specific problem types, such as migration. In this case, we have a researched case (Bolivia) and an un-researched case (Tanzania). The question here is, what can we learn (if anything) from migration issues on climate change in Bolivia for Tanzania? Similarly, we can discuss the same situation in another problem, such as droughts, looking to transfer knowledge about droughts and climate change from the known case (the Sahara) to an un-researched region, such as Siberia. In both situations, the transfer of knowledge takes place within each problem type.

In the second instance b), we consider a situation where we want to learn about whether a policy or measure to address migration also applies in addressing droughts in the context of climate change, as is often the case when seeking to mainstream adaptation policies to address multiple adaptation problems. Here we can take knowledge on what we know works for migration in one context and apply this to address drought issues in another context. For example, we could ask: what could we learn, if anything, about how climate change migration issues in Bolivia that could be combined and/or inter-related to issues of climate change and droughts in Siberia?

In both situations, there are assumptions made about the extent of transferable case study knowledge both between units of the same problem type and between units of different problem types, where the question remains: under what conditions can we transfer knowledge between inter and intra-problem types? We concur with Krohn (2010) in arguing that adequately transferring knowledge across cases, as opposed to generalizing findings, is a crucial yet neglected methodological challenge. This is an important issue to overcome methodologically, given that simply reporting on ‘what works?’ in a given context is not sufficient knowledge in itself for practical application elsewhere, especially if this is devoid of complementary knowledge that also answers: ‘for whom did it work? and how?’ (Pawson 2006).

Challenges associated with the transfer of knowledge across diverse and context-specific cases have been the subject of discussion and elaboration in other research communities that have developed various kindred concepts for learning from case studies. Those discussions provide suggestions for structuring key considerations for knowledge transfer across cases in TD research. Here, we mention just some of those. For instance, community-based climate change adaptation uses the concept of scaling out pilots, i.e. isolated localized examples of adaptation, for wider geographical application, while highlighting as a core challenge that local specificities, e.g. success factors in one community, may not be transferable to another community (Gogoi et al. 2014). In much in the same way, Burdack et al. (2014) discuss the applicability of their findings from a case study on water-rights trading for managing water demand and supply to other regions by highlighting the contextual factors that apply in Australia for this intervention to work with the desired effects in that context. In policy sciences, indicators for diffusion of innovations are discussed to supplement the information gained in local or regional case studies. Determining valid indicators requires a systematic investigation of conditions under which a measure may hold or not (Brunner 2014; Lasswell 1971). Therefore, the community-based approach and policy sciences both consider conditions for or against transferability of transformation knowledge.

Transition management, using local or regional transition experiments to explore the dynamics of transitions in societal systems, takes a broader approach. Core concepts in transition management are deepening, broadening and upscaling of transition experiments used for analyzing and managing both the process and the substance of a successful transition experiment in sustainable development (van den Bosch 2010, p. 74ff). Deepening is about learning from a project in its context, while scaling-up is about embedding the transition experiment in dominant ways of thinking, doing and organizing. Broadening, i.e. replicating and linking to other contexts and functions, comes to some respect closer to what we mean by transfer in this paper, i.e. applying knowledge to other cases. However, broadening is different in that it stresses variation and recombination of elements. In van den Bosch (2010), the basic mechanism of broadening is in conducting different experiments in a variety of contexts, either to get the new or different social structures or practices applied in a variety of contexts, or enrich the social structures or practices (van den Bosch 2010).

In philosophy of science, there are several systematic analyses and proposals. For instance, Bengtsson and Hertting (2014) propose that empirical findings are portable from one context to other contexts, if they can be related to ideal-type patterns of action on a more abstract level, and that can function as the vehicle for transfer. Also, in realist evaluations, the ‘context–mechanism–outcome' model (C–M–O) combines the empirical and the conceptual level for considering transferability of knowledge, arguing that the configuration of context, mechanism, and outcome need to be considered in order to judge what works for whom and in under what circumstances (Pawson 2006, p. 25). Cartwright proposes to use the concept of INUS conditions to analyze conditions for transferability of knowledge to a different case. Transferability is given if all the required supporting factors are in place. A supporting factor conceived as an INUS condition is an “Insufficient but Necessary part of an Unnecessary but Sufficient condition for getting a contribution to the effect you want” (Cartwright and Hardie 2012, p. 63).

We find in these discussions of kindred approaches that a common feature regarding transferability of knowledge centers on conditions for transferable lessons from one case to another, rather than just the outcomes. Hence, a general answer to the question we pose, ‘under what conditions can co-produced knowledge be transferred to another case?’ seems to be simple: it depends on whether the cases in question are sufficiently similar in relevant aspects, while not dissimilar in other relevant aspects. Therefore, we propose to conceptualize transferring knowledge across cases as arguments by analogy. Arguments by analogy are widely used in everyday life as well as in science. They can serve discovery or justification, or play a programmatic role in the development of a field (Bartha 2013). We focus on their justificatory role. Arguments by analogy are non-deductive inferences, which means that they are risky. In order to assess the plausibility or strength of analogical inferences from a source to a target, one has to judge whether source and target are sufficiently similar in the relevant regards and do not show important dissimilarities. However, there are no simple, general and strict rules to answer these questions, since answers have to rely on the substance of the problem and the context, where the problem is addressed. To our knowledge, there is neither much discussion on requirements and strength of analogical inferences for asserting transfer of knowledge across cases in TD research methodology, nor do we see (yet) empirical TD research that provides grounded answers to these questions.

We find that a necessary starting point for investigating transferability of knowledge across cases in TD research is to first account for the perspectives of those involved in a TD research context on issues of transferability. However, we also caution on two challenges for dealing with transferability across cases that TD researchers need to consider. On the one hand, the diversity in contexts and specific case-based results typical of TD research could lead to an ‘ideographic trap’ because each case study is regarded as unique and transferability of knowledge seems impossible or irrelevant (Gallati and Wiesmann 2011). On the other hand, knowledge could be transferred to other case studies based on mere assumptions, or on implicit but diverging use of considerations about relevant similarities and dissimilarities. However, inconsistent practice cannot justify and provide assurance for transfer from one case to another. If researchers and policymakers in TD collaborations do not deliberately consider conditions for transferability and eventually find themselves misled in doing so, they risk that the quality of their research on cases is questioned. For instance, as calls for auditability of quality appear to proliferate, inconsistent evidence is perceived as one pertinent quality problem in science for policy (Bilotta et al. 2014; Boyd 2013; Gluckman 2014). With this problem in mind, the question of how to conceive and judge transfer of knowledge across cases, and how transferability of knowledge is to be distinguished from generalizability of findings, requires a closer look.

## Shortcomings of the conventional approach to evidence-based policy from a TD perspective

A common critique towards TD case study research is that it does not provide generalizable results, as is the case through other approaches such as randomized controlled trials (RCTs). Along this line, evidence-based policy is an increasingly influential concept, originally developed in the field of health for clinical trials (Dobrow et al. 2004; Elphick and Smyth 2004) and now also used in research for sustainable development (Bilotta et al. 2014; Holmes and Clark 2008; Pullin and Knight 2009). In evidence-based policy, results from RCTs are considered the gold standard of evidence for policy. RCTs test the significance of statistical relations between variables. Only if a broad range of possibly influential factors in the real world is excluded, can observed frequencies on a few variables under standardized conditions allow for statistical tests for inference on whether some functional or causal relation holds in general. We refer to evidence-based policy as using just the evidence from RCTs as the reliable scientific basis for policy advice, as to the conventional approach of evidence-based policy. RCTs may provide valuable abstract information for structuring a policy problem, if used together with additional information that corrects idealization and accounts for the context of application (see “Methodological implications of conceptualizing transfer of knowledge across cases in TD research as analogical arguments”). However, the conventional approach to evidence-based policy ignores the fact that RCTs test abstract relations, assuming that these relations would hold more or less in the same way in concrete contexts. Hence, the expectation that the conventional approach to evidence-based policy will be implemented and bring about the intended effects has not been fulfilled in many cases.

For policy to be implemented in a given context, it is required that “the information is perceived by relevant stakeholders to be not only credible [i.e. based on scientific evidence], but also salient and legitimate” (Cash et al. 2003, p. 7). Information is salient if at the time given it is considered relevant by policymakers. Information is legitimate if the way it is produced takes account of “stakeholders’ divergent values and beliefs” (Cash et al. 2003, p. 7). As Cash et al. (2003) highlight, there are fundamental trade-offs between salience, credibility and legitimacy, since, to some extent, accounting for salience and legitimacy in producing credible results contradicts the methodological requirements of standardized approaches to idealized problems abstracting from many features of the concrete cases investigated, as in RCTs.

From a TD perspective, a first criticism relates to inadequate specification and application of criteria. Conventional approaches in evidence-based policy do not consider how and by whom scientific evidence is interpreted for a particular policy problem (Dobrow et al. 2004; Holmes and Clark 2008; Howick et al. 2013). However, policymakers’ perspectives are key for legitimacy, credibility and salience. Taking the IPCC process as an example, interpretation of evidence regarding legitimacy and salience is first done by scientists when writing the Assessment Reports. Interpretation by policymakers follows in the IPCC plenary towards the end of the knowledge production process. From a TD perspective, considerations of legitimacy and salience take place too late in the IPCC process, since perspectives of policymakers need to be accounted for when establishing the evidence. In co-production of knowledge, policymakers are included in the first stage of problem framing, ensuring that the questions addressed by research will be relevant, i.e. salient, and results credible, i.e. evidence appropriate for the particular policy problem (Wiesmann and Hurni 2011). Contrary to basing evidence-based policy on RCTs alone, the starting point of basing evidence-based policy on TD research is in establishing both the evidence for scientific information and its salience, based on legitimacy for a particular context (Pohl and Hirsch Hadorn 2007; Wiesmann et al. 2008). From identifying, framing and structuring the problem, TD research strives to account for perspectives and knowledge requirements of policymakers, since credibility of results and their salience, i.e. relevance needed to account for legitimacy, largely influence their stakes in the particular policy problem.

A second criticism that evidence-based policy based on RCTs faces from a TD perspective is that there are several criteria for quality. For instance, there is broad agreement that evidence has to be assessed differently in basic and applied research. For basic research, it is important to minimise the risk of Type I errors in RCTs, i.e. a false positive (claiming an effect when there is no effect). When scientific evidence is used to inform policy on real-world problems, however, minimizing the risk of Type II errors in RCTs (claiming there is no effect when there is one) becomes more important because of the precautionary principle that prioritizes possible negative impacts on human beings and the environment. Kriebel et al. (2001) add Type III errors, where scientific evidence produced in well-defined and controlled research environments is used to inform ill-defined ‘wicked problems’. Wicked problems (Rittel and Webber 1973) cannot be definitively described, lack clarity on which and whose values are involved and do not allow for a single, definitive and optimal solution. Therefore, Type III errors link back to the question of how and by whom scientific evidence is produced and interpreted for a particular policy. Consequently, accounting for these requirements speaks for TD case study research and against the conventional approach to evidence-based policy.

## The specific challenges of transfer of knowledge across cases for TD research

**Fig. 2:**
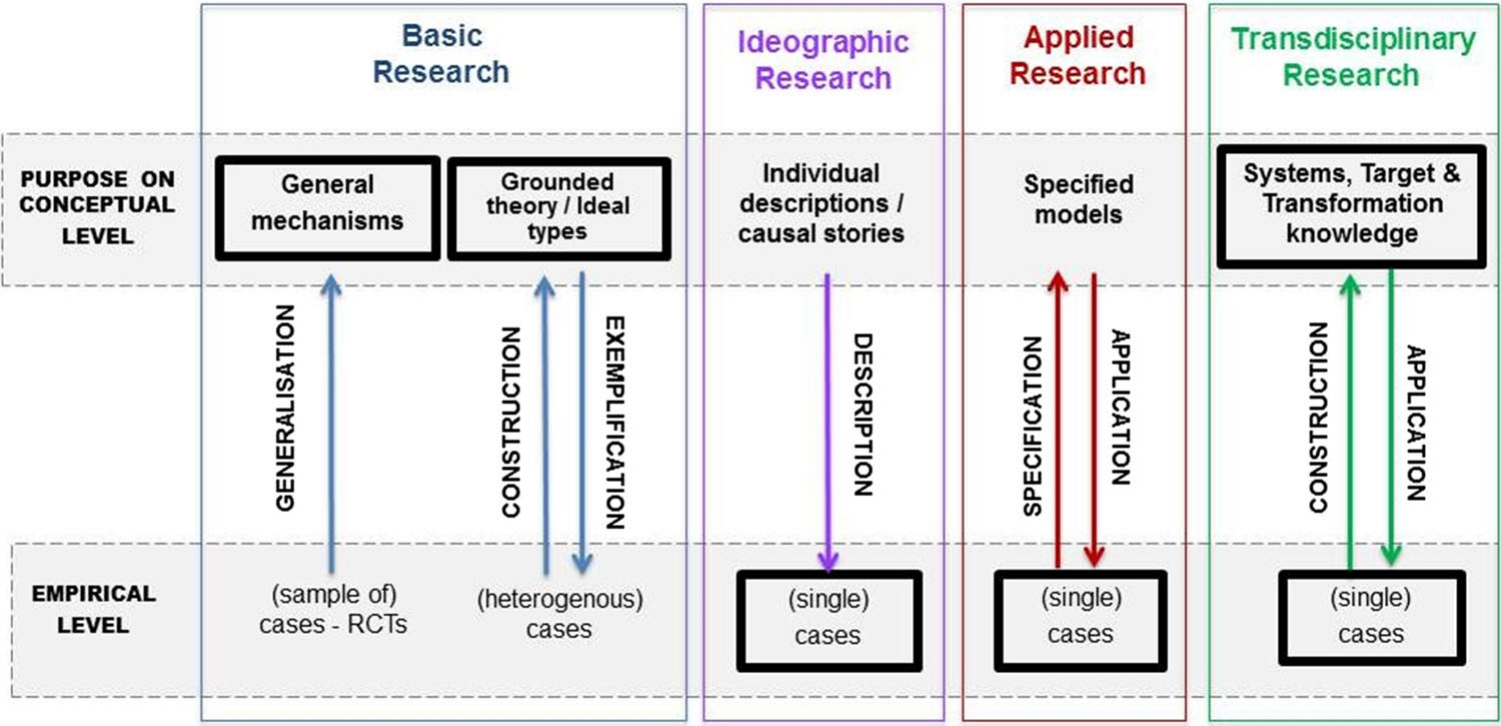
Functions of cases in TD research compared to other forms of conducting research

There are many ways for how cases are used in research. If a case is understood as an empirical manifestation of a phenomenon to be investigated (Gerring 2007, p. 19), then all empirical research can be said to investigate cases in some way. However, depending on the purpose and paradigm of the research, criteria to select cases, their functions and the methods used, differ (Hirsch Hadorn 2017). To clarify the specific challenges for transferability of knowledge from TD case study research across cases or problems, we compare it to the perspectives on investigating cases in basic standardized research, grounded theory, applied research and ideographic research (see Fig. 2). We conceive these perspectives as ideal–typical simplifications in order to better highlight their specific characteristics (Weber 1962), while in research practice, several perspectives may overlap or be combined. For each perspective on investigating cases, we distinguish (a) the empirical level at which characteristics of cases are observed, (b) the conceptual level to structure the information on cases for the purpose in question and (c) how both levels relate. The relations between the empirical and the conceptual level determine what can be learned from investigating cases and respective requirements for transferability. These relations in turn are determined by the underlying paradigm and the purpose of research.

### Basic research

Basic research can be conducted under a standardized or a grounded theory perspective. Finding general rules in order to explain and predict natural and social processes is rooted in positivism or post-positivism (Guba and Lincoln 2005). To find such rules, experiments are designed that test hypotheses by quantitative methods. Such research typically refers to randomly selected cases as empirical instances (be it in the real world or the laboratory) that exhibit certain properties in order to measure and analyze how these properties are distributed and correlated among a standardized set of cases. Results are taken as evidence for or against a general description or explanation of how properties depend on each other and, therefore, are transferable to cases that have not (yet) been investigated. RCTs use this paradigm of generalizable rules. Strictly speaking, treating cases in this way does not align with a ‘case-study’ label or characterization, since the scientific interest is not on the cases, although properties of cases provide the evidence on a statistical level.

### Applied research

Applied research is typically conceived as the application of concepts, methods and models from basic research to a specific case (Baumgärtner et al. 2008). Strictly speaking, it does not stand on its own but builds on basic research concerning the theoretical level, while its main interest is on the empirical level and on the specific cases themselves. Abstract models and concepts from basic research are adapted and used to describe, predict or manage the concrete problem situation at hand. If an empirical situation is classified as a case for a certain type of problem, then transferability of knowledge across cases can be judged by whether this classification is correct for the cases under consideration.

### Grounded theory

Grounded theory (Glaser and Strauss 1967) is used in the social sciences to build theory based on qualitative analysis of contrasting cases. It aims at better understanding the heterogeneity of different phenomena or supporting practitioners in dealing with a phenomenon. Cases are used as empirical basis to ground the construction of theories (Walton 1992) or ideal-types (Hirsch Hadorn 1997). Since comparative analysis is key, a good sample is made up of heterogeneous cases rather than by a large number of cases. To the extent that empirical cases instantiate the relevant features of a theory or ideal-type, this assures classification and consequently transferability across cases of the same type.

### The ideographic approach

The ideographic approach (Guba and Lincoln 2005) takes empirical cases as subjects of interest in themselves. The purpose of ideographic research is to describe the individual composition of features in single (actual or historical) real-world events or processes in order to understand concrete phenomena and their story, how they came about and what came afterwards. These phenomena are of interest in a specific socio-historical context and the values and beliefs held there; thus the question of transferability is not meaningful for ideographic research.

### TD case study research

TD case study research does not fully fit into any one of these four perspectives. Instead, TD research combines features from several of them. The real-world situation under investigation is a subject of interest on its own, like in ideographic research. The purpose is to develop knowledge for use to change the specific situation, like in grounded theory or applied research. However, the problem(s) to be addressed in a concrete context are not simply predefined by general models for further specification, as in applied research, but open to discussion and determined in joint problem structuring. Joint problem structuring includes ideographic elements to understand the specific combination of features of the real-world situation in relation to what is at stake, and for whom. However, to provide a basis for transferability of knowledge across cases, constructing a model of why knowledge works (or not) in this case is also needed. For an example on how knowledge transfer across cases has worked, see Box 1. While these models may integrate knowledge about general relations, their purpose is not to enable general inferences, since this must not be done on the basis of single-case and small-*n* studies (Bengtsson and Hertting 2014). Instead, models constructed in TD case study research should be used to identify the conditions that speak for or against the effectiveness of knowledge for policy if transferred to another case.

## Methodological implications of conceptualizing transfer of knowledge across cases in TD research as analogical arguments

From a methodological standpoint, as discussed in “Framing the problem and current practice”, evaluating transferability across cases can be conceived as assessing the plausibility of an argument by analogy. Arguments by analogy refer to relevant similarities of cases in order to justify an inference from one case (the source) to a different case (the target). To specify this vague conceptual idea, we rely on Bartha (2013)’s discussion of analogical arguments and his review of general common sense guidelines for evaluating analogical arguments discussed in argumentation theory.^1^

In an argument by analogy, we typically do not have a one-to-one mapping between all elements, properties, relations and functions in the source, on the one hand, and in the target on the other. Therefore, an inference by analogy is a non-deductive inference. While deductive arguments are correct if they conform to some formal schema, this is not possible for non-deductive inferences, since those are risky. Non-deductive inferences can be assessed by how strong or plausible they are. This depends on whether all the relevant information is considered in the premises of the argument. Hence, the plausibility of such an inference results from whether all the relevant similarities and dissimilarities between source and target have been identified, and how perceived similarities and dissimilarities are weighed against each other. Bartha (2013) lists the following common sense guidelines (G), based on a review of the literature in argumentation theory:

- (G1) The more the similarities (between two domains), the stronger the analogy.
- (G2) The more the differences, the weaker the analogy.
- (G3) The greater the extent of our ignorance about the two domains, the weaker the analogy.
- (G4) The weaker the conclusion, the more plausible the analogy.
- (G5) Analogies involving causal relations are more plausible than those not involving causal relations.
- (G6) Structural analogies are stronger than those based on superficial similarities.
- (G7) The relevance of the similarities and differences to the conclusion (i.e. to the hypothetical analogy) must be taken into account.
- (G8) Multiple analogies supporting the same conclusion make the argument stronger.

Clearly, these guidelines are still individually quite vague, also on how to apply them collectively. Hence, they do not work as algorithms that determine the result. However, they can still be useful since they provide guidance for reasoning. This is how Chow characterizes heuristics in general, namely as “satisficing cognitive procedures that can be expressed as rules one reasons in accordance with” (Chow 2015, p. 1005). Hence, guidelines for weighing similarities and dissimilarities can be used as heuristics to evaluate transfer across cases. Whether the guidelines discussed by Bartha are appropriate for TD research, is the subject of further empirical work.

While these guidelines are useful, they are not sufficient for assessing analogical inferences, since their plausibility also depends on material information. In the context of TD research, the fact that analogical arguments cannot be assessed by referring to some formal schema that would inform about its correctness, is not a weakness but an advantage. In assessing an argument by analogy, one has to clarify which of the many items such as elements, properties, relations or functions are relevant for the inference to be assessed. Items count in evaluating transferability, if similarity or dissimilarity of source and target with respect to these items strengthens or weakens the analogical inference. Learning about relevance of items is not a formal but a material question that depends on empirical information about the specific problem at hand. At this point, accounting for the characteristics of TD research is crucial.

For instance, when assessing transferability of transformation knowledge developed in TD research, one has to consider how this transformation knowledge is embedded in the specific knowledge about the target and about the system (Pohl and Hirsch Hadorn 2007).^2^ As Barzelay (2007) highlights, transfer of knowledge from a source to a target “is more complex than ascertaining whether a given practice is effective in source sites, as evaluation researchers might have it; it requires theoretical insight into how observed practices actually mobilize human action and bring about substantively significant effects” (Barzelay 2007, p. 522). When looking for proposals on how one can learn about which items would count for transfer, we found that several scholars have developed heuristics, i.e. guidelines for how to investigate those items. Barzelay proposes an explanatory heuristic similar to “restrictions and options” (Hirsch Hadorn et al. 2002), where researchers can investigate practices in source sites to prepare the ground for what he calls extrapolation of practices from source to target sites. An iteration between implementing changes, observing, and planning new interventions based on the observations is what strategies such as real-world experiments (Gross et al. 2005) and adaptive governance (Brunner 2010) suggest. Both can be used as strategies to learn from implementation in different contexts about causal relevance of particular aspects, such as conditions of successful transfer of knowledge (Bengtsson and Hertting 2014; Gerring 2007). Cartwright and Hardie (2012), elaborate on a framework and principles for knowledge transfer in evidence-based policy that is not conventional evidence-based policy. They suggest thinking of a complex array of factors to be considered for knowledge transfer. Individual factors relevant for knowledge transfer across cases are “an insufficient but non-redundant part of a complex of factors that are unnecessary but together sufficient” (Cartwright 2012, p. 979). The factors may operate on concrete or abstract levels and may be complemented by additional supporting factors. Some accurate general claim based on RCTs may be part of this complex condition but must not in itself count as sufficient to warrant effectiveness for reasons discussed in previous sections.

For our problem, i.e. how to assess transferability of knowledge across cases in TD research, what is needed most is guidance for how to answer the following empirical question: which items in a given transdisciplinary case study count for transferability of knowledge across cases? As in the case of guidelines for weighing similarities and dissimilarities (Bartha 2013), a structured set of criteria would be helpful. As part of this structure, one might think of distinguishing not only between forms of knowledge (transformation, systems and target knowledge), sources of knowledge (academic disciplines and stakeholders and practice experts), but also between substantive and procedural knowledge most important in TD research. Such criteria could provide an additional heuristic to address the material aspects of relevance in assessing the strength of arguments by analogy when transferring knowledge across cases. In so doing, this additional heuristic could guide the analysis of effectiveness in the proposed solutions through diverse, variable and complex conditions in the given cases.

However, as Crasnov (2012) has pointed out with reference to political science, relating different sorts of evidence in a mixed methods approach, when judging effectiveness of outcomes in concrete cases, is still a debated issue. A first step to improve this situation would be if researchers would explicitly discuss what knowledge, i.e. lessons from their own case study, could be reasonably transferred to other cases and for which reasons. Efforts to systematize transferability of knowledge across cases would benefit from such empirical information, to provide an evidentiary basis for structuring quality in TD research by means of criteria to be used as heuristics.

## Summary and conclusion

There is quite a way to go until a structured approach to knowledge transfer across cases in TD case study research is developed. The problem of transferability of knowledge across diverse and context-specific cases is discussed in various fields. In this paper, we argue that the problem needs to be conceptualized in a way that accounts for the particular requirement of TD research, i.e. an approach that deals with cases where knowledge is co-produced by teams of researchers and stakeholders. Therefore, it is not only different from basic, but also from applied and ideographic research.

In summary, we propose to conceptualize the problem of transferring knowledge across cases as arguments by analogy. Hence, we suggest a consideration to handle transferability of knowledge from TD case study research across cases, regarding whether the cases in question are sufficiently similar in relevant aspects while not dissimilar in further relevant aspects. On the one hand, this approach calls for explicit material considerations needed to learn about which aspects of cases are relevant. What makes appraising transferability of knowledge across cases in TD research special is the fact that relevant aspects include what teams of researchers and stakeholders may take as necessary, sufficient or supporting factors for concrete cases. In addition, lessons learned from TD case studies are not only restricted to the substance or content-related matter, but may also include knowledge about processes employed for knowledge co-production. On the other hand, formal considerations on how to weigh perceived relevant similarities and dissimilarities of the cases at hand for transferability of knowledge are needed. Here, TD research can build on the literature in argument analysis as a starting point.

We have argued that transfer between cases in TD research must be distinguished from generalizing across cases. Transfer across cases is conceptualized as an analogical inference that is assessed regarding its strength or plausibility by investigating the relevant similarities and dissimilarities between the cases at hand and weighing them. Generalizing from cases is conceptualized as a statistical inference that is assessed regarding its inductive risk, through approaches such as RCTs. Not clearly distinguishing between these different types of inference and their preconditions opens a door to unjustified interpretations of results.

However, there are few empirical examples of how these problems are dealt with in practice, even though this is precisely the sort of information that yields insights on how problems of transferability across cases are addressed in context. We assume that the transfer of knowledge from one case to another is often done on implicit assumptions, since a systematic conceptualization and an easy-to-handle method for explicit considerations is missing. Currently we know little about knowledge transfer across cases in TD case study research. For instance, we do not know whether knowledge is transferred (if at all), what kind of knowledge it is (e.g. about facts, about processes), whether researchers and stakeholders differ in what they transfer, and what kind of considerations to transferability they give, if any at all. In an SNF funded project (2016–2018),^3^ we analyse the following three research questions: (1) what knowledge do researchers and stakeholders transfer across cases, if at all? (2) What considerations do researchers and stakeholders apply when transferring knowledge across cases? (3) Collectively, what typical considerations for transfer of knowledge across cases exist in TD research? Based on a qualitative analysis (interviews, document analysis and informal exchange) of a heterogeneous sample of 12 TD projects in the field of global environmental change, we will provide some answers to these questions. However, a concerted effort in the TD research community is still needed to fill this empirical gap, by making explicit the considerations taken by knowledge producers on transferability. These results would allow for a grounded exploration of possible methodological advances and enable a systematic structure to emerge for considering effectiveness of policy options based on TD research.

## Box 1: Lessons on transferability from a long-term transdisciplinary research activity in the Mount Kenya region

The vast region stretching northwards from tropical Mount Kenya to semiarid lowlands is characterized by steep ecological gradients, fast socio-economic transition and rapid land use transformation. Transformations towards sustainable development in this dynamic setting have to deal with wicked problems such as high poverty levels, increasing economic disparities, rapidly degrading environmental functions and increasing upstream–downstream conflicts. The interrelation between these problems and their factual uncertainty, value loads and conflicting interests between multiple stakeholders pose major challenges for sustainability-oriented efforts and supporting research, requiring a transdisciplinary approach.

An interdisciplinary team of Kenyan and Swiss researchers has taken up the challenges of TD research for more sustainable development in this complex setting. In various constellations and under varying project umbrellas the team is engaged in the region since more than three decades. As it is common for engaged transdisciplinary endeavors, that the driving force of research is not to aim at ‘just another case study’ for generalization purposes, but to substantially contribute to more sustainable development in this very context and to the well-being of its half a million people. The contextual orientation implies that in such transdisciplinary endeavors, questions of transferability are subordinate in the research design to questions such as adequate recursive and iterative research approaches at the concrete science-society interfaces.

However, the analyses of the history and development of the long-term transdisciplinary involvement for sustainable development in the Mount Kenya region indicates quite some transfer of springs. The following are examples of such transfers of results and outcomes of various different kinds to other cases be these of the same or of a different type:

- Transfer of disciplinary insights, e.g. in the fields of climate change or peasants’ adaptation strategies where research in the Mount Kenya region contributed profound case studies to disciplinary development;
- transfer through replication of approach and studies, e.g. the replication of the integrated transdisciplinary approach in the Kilimanjaro region where similar sociocultural competitions are met;
- transfer of conceptual and theoretical advancements, e.g. theoretical contributions to the global sustainability debate or contributions to principles of intercultural and transdisciplinary research partnerships derived from the long-standing experiences in the Mount Kenya region;
- transfer of methodological innovations, e.g. the recognition of typical configurations or patterns of problems and potentials for sustainable development that can be clustered into syndromes or archetypes;
- transfer of innovation adoption pathways, e.g. conditions for the uptake of an innovative and socially adapted weir for river water regulation in other contexts;
- transfer of policies, e.g. consolidation of regulations in national legislations developed and tested in the region on camel milk and its marketing as an important component of pastoralist livelihoods; and
- transfer of governance structures, e.g. the spread of grassroots water users’ associations and their bylaws that were originally initiated in the region watersheds in East Africa and their reflection in water policy reforms at national and transnational level.

From the above examples, two questions arise: first, can transfer outcomes of transdisciplinary endeavors be classified or clustered into distinctive categories and can such categorization help in designing transdisciplinary projects and programmes? Second, what conditions and measures promote successful transfer within the identified transdisciplinary project undertaking? Answering these two questions could significantly increase the impact and relevance of transdisciplinary research and is the core of studying transferability (Kiteme and Wiesmann 2008, Ehrensperger et al. 2015).

## Footnotes

1 Bartha, in his treatment of analogy and analogical reasoning, also refers to approaches of analogical reasoning in philosophy of science. For instance, he discusses Mary Hesse’s theory and material criteria for analogical inferences from the model to the target in physics, or Kuhn’s practice of analogical reasoning in his case studies on the history of physics and chemistry, which Kuhn used for the discovery and justification of his theory of scientific revolutions. An extensive treatment of analogical reasoning in model construction and inferences to the target for a broad range of sciences is discussed in Creager et al. (2007). While it is interesting to see the importance of analogical reasoning in the epistemology of modelling today, it is important to acknowledge that TD research provides a different framework for analogical reasoning for transfer of knowledge across cases.

2 Because transformation, systems and target knowledge are specified in relation to each other, transfer of knowledge has to account for relevant inter-dependencies between the forms of knowledge in the cases at hand. Despite of this fact, each form of knowledge comes with requirements of its own that need to be considered. Target knowledge, for instance, requires debate among those involved for proper specification of the vague goals and principles of sustainable development and agreement on legitimate trade-offs among them to work as concrete targets that can be addressed in a TD project. However, a systematic treatment of transferability of systems, target and transformation knowledge as different forms of knowledge is beyond the scope of this paper and would need elaboration in a separate study.

3
http://p3.snf.ch/Project-162781.
